# Clinical Significance, Species Distribution, and Temporal Trends of Nontuberculous Mycobacteria, Denmark, 1991–2022

**DOI:** 10.3201/eid3009.240095

**Published:** 2024-09

**Authors:** Victor Naestholt Dahl, Andreas Arnholdt Pedersen, Anders Norman, E. Michael Rasmussen, Jakko van Ingen, Aase Bengaard Andersen, Christian Morberg Wejse, Troels Lillebaek

**Affiliations:** Statens Serum Institut, Copenhagen, Denmark (V.N. Dahl, A. Norman, E.M. Rasmussen, T. Lillebaek);; Aarhus University Hospital, Aarhus, Denmark (V.N. Dahl, C.M. Wejse);; Aarhus University, Aarhus (V.N. Dahl, C.M. Wejse);; Odense University Hospital, Odense, Denmark (A.A. Pedersen);; Lillebaelt Hospital, Vejle, Denmark (A.A. Pedersen);; Radboud University Medical Center, Nijmegen, the Netherlands (J. van Ingen);; Rigshospitalet, Copenhagen (A.B. Andersen);; University of Copenhagen, Copenhagen (T. Lillebaek)

**Keywords:** nontuberculous mycobacteria, tuberculosis and other mycobacteria, bacteria, clinical relevance, pathogenicity, public health surveillance, epidemiology, incidence, Denmark

## Abstract

Nontuberculous mycobacteria (NTM) are emerging as notable causative agents of opportunistic infections. To examine clinical significance, species distribution, and temporal trends of NTM in Denmark, we performed a nationwide register-based study of all unique persons with NTM isolated in the country during 1991–2022. We categorized patients as having definite disease, possible disease, or isolation by using a previously validated method. The incidence of pulmonary NTM increased throughout the study period, in contrast to earlier findings. *Mycobacterium malmoense*, *M. kansasii*, *M. szulgai*, and *M. avium* complex were the most clinically significant species based on microbiologic findings; *M. avium* dominated in incidence. This study shows the need for surveillance for an emerging infection that is not notifiable in most countries, provides evidence to support clinical decision-making, and highlights the importance of not considering NTM as a single entity.

Nontuberculous mycobacteria (NTM) are emerging as important causative agents of opportunistic infections globally (*1*). NTM can cause a wide spectrum of disease, leading to various clinical manifestations depending on the exposure, species virulence, host immunity, and site of infection (*2*). Clinical management and studies of pulmonary NTM infections are often challenged by difficulties in discriminating between transient NTM findings in nonsterile body sites after environmental exposure (often referred to as colonization, contamination, or isolation) as opposed to true NTM disease, because positive NTM cultures do not necessarily reflect disease.

For NTM pulmonary disease, the diagnostic criteria are based on the presence of symptoms along with radiologic and microbiologic findings (*3*). Although those criteria have been widely implemented, the application still relies on the individual clinician’s subjective assessment and decision. As an alternative strategy, previous studies have validated infections by using microbiologic data only as a proxy for clinically significant pulmonary NTM disease, dividing NTM into categories reflecting the likelihood of disease (*4*–*6*). In Denmark and elsewhere, data on the clinical significance of NTM, including specific NTM species, is sparse. Consequently, in this nationwide study, we examined the clinical significance, species distribution, and temporal trends of NTM isolates over 32 years.

## Methods

For decades, mycobacterial diagnostics and surveillance in Denmark have been centralized at the International Reference Laboratory of Mycobacteriology (IRLM) at Statens Serum Institut in Copenhagen. IRLM stores nationwide data and cultures from patients tested for mycobacterial infections as the only such laboratory in the country. In this nationwide register-based study, we combined mycobacteriologic data from IRLM with population data from Statistics Denmark (https://www.dst.dk).

We evaluated the clinical significance of NTM isolates in Denmark by using only microbiologic data for all persons in Denmark who had >1 NTM isolate identified during 1991–2022. We only counted an incident case of NTM once and thereafter excluded the affected person from the population at risk for NTM infection. We excluded from our study patients with *Mycobacterium gordonae* isolates only, because that species is considered a clinically irrelevant contaminant (*6*,*7*). We also excluded patients with multiple NTM isolated concomitantly.

As part of routine diagnostics, samples were cultured on liquid media with BACTEC MGIT 960 (BD Diagnostic Systems, https://www.bd.com) and on solid Löwenstein–Jensen slants (Statens Serum Institut Diagnostica, https://ssidiagnostica.com) for up to 8 weeks. Throughout the study period, NTM species were identified using different methods, including AccuProbe (Gen-Probe, https://www.hologic.com) and supplementary biochemical tests during 1991–2001, InnoLipa Mycobacteria version 2 (InnoGenetics, https://www.fujirebio.com) during 2001–2012, GenoType Mycobacterium CM/AS (Hain Lifescience, https://www.hain-lifescience.de) during 2012–2022, and GenoType NTM-DR (Hain Lifescience) during 2016–2022. Supplementary species identification also was used, targeting the 16S (2006–2022) and internal transcribed spacer (2015–2022) regions with Sanger sequencing.

Using a previously validated method and modifying guideline criteria (*3*), we categorized patients as having either definite disease, possible disease, or isolation (*4*,*5*). For pulmonary NTM, including gastric lavages, we defined definite disease as either >3 positive samples, 3 positive samples including >1 obtained by bronchoscopy or pleurocentesis, or >1 positive sample from a lung or transbronchial biopsy. We defined NTM isolation as only 1 positive NTM sample and categorized the remaining patients as having possible disease. We defined all patients with a positive NTM sample from an extrapulmonary location as having definite disease, except for patients with only 1 sample of urine or feces, who we categorized as possible disease. We categorized patients with samples from both pulmonary and extrapulmonary locations as having disseminated disease. We grouped species by using phylogenetic classifications described by Tortoli et al. (*7*). As we considered the discrimination of NTM species identification within a complex or group to have changed much over time, we grouped NTM species for main analyses.

We compiled descriptive statistics by using counts and percentages for categorical data and medians and interquartile ranges (IQRs) for continuous data. As an illustration of the clinical significance of pulmonary NTM, we calculated the number and corresponding percentage of patients fulfilling the modified disease criteria by each species for definite disease only and for definite and possible disease combined. We calculated annual incidence rates (IRs) as the number of new patients with NTM in the numerator and the total number of patients in Denmark in the denominator for the given year. We evaluated trends over time by using a Poisson model with age groups, sex, and calendar year as explanatory variables, and we calculated IRs by using the population of Denmark as response variables. We conducted statistical analyses and generated figures by using R version 4.2.3 (The R Project for Statistical Computing, https://www.r-project.org). The study was approved by Denmark’s Data Protection Agency through the Department of Compliance at Statens Serum Institut, Copenhagen (approval no. 22/00845).

## Results

A total of 4,123 unique patients had a positive NTM culture, excluding 896 with *M. gordonae* and 34 with multiple species isolated concomitantly, equaling 129 new patients annually on average. Median patient age was 59 years (IQR 33–72 years); 1,977 (48%) were female and 2,146 (52%) male, and 3,673 (89%) were born in Denmark.

Most samples were pulmonary (2,851 [69%]), whereas approximately one fourth were extrapulmonary (1,106 [27%]); few patients had disseminated NTM (166 [4.0%]) (Table). The most frequent isolates were *M. avium* complex (MAC) (2,547 [62%]), whereas other species were seen much less frequently. *M. avium* (2,046 [80%]) and *M. intracellulare* (334 [13%]) accounted for most cases of MAC.

**Table:** Disease localization, species distribution, and clinical significance for patients with nontuberculous mycobacteria, Denmark 1991–2022*

Characteristic	Total	Years			
		1991–1998	1999–2006	2007–2014	2015–2022
No. patients	4,123 (100)	948 (100)	793 (100)	1,027 (100)	1,355 (100)
Disease localization					
Pulmonary	2,851 (69)	495 (52)	523 (66)	769 (75)	1,064 (79)
Extrapulmonary	1,106 (27)	341 (36)	255 (32)	242 (24)	268 (20)
Disseminated†	166 (4.0)	112 (12)	15 (1.9)	16 (1.6)	23 (1.7)
*Mycobacteria* species group‡					
*M. avium* complex	2,547 (62)	634 (67)	433 (55)	592 (58)	888 (66)
* M. avium*	2,046 (80)	575 (91)	370 (85)	467 (79)	634 (71)
* M. chimaera*	119 (4.7)	0	0	2 (0.3)	117 (13)
* M. intracellulare*	334 (13)	59 (9.3)	62 (14)	114 (19)	99 (11)
Other or unspecified	48 (1.9)	0	1 (0.2)	9 (1.5)	38 (4.3)
*M. abscessus-chelonae* complex	275 (6.7)	49 (5.2)	50 (6.3)	86 (8.4)	90 (6.6)
*M. xenopi* group	231 (5.6)	37 (3.9)	45 (5.7)	52 (5.1)	97 (7.2)
*M. fortuitum-smegmatis* group	202 (4.9)	57 (6.0)	48 (6.1)	34 (3.3)	63 (4.6)
* M. malmoense*	168 (4.1)	38 (4.0)	45 (5.7)	54 (5.3)	31 (2.3)
* M. marinum*	128 (3.1)	23 (2.4)	25 (3.2)	41 (4.0)	39 (2.9)
*M. celatum* group	117 (2.8)	13 (1.4)	19 (2.4)	45 (4.4)	40 (3.0)
* M. kansasii*	81 (2.0)	11 (1.2)	12 (1.5)	32 (3.1)	26 (1.9)
*M. simiae* complex	43 (1.0)	11 (1.2)	10 (1.3)	15 (1.5)	7 (0.5)
* M. parascrofulaceum/scrofulaceum*	40 (1.0)	10 (1.1)	3 (0.4)	10 (1.0)	17 (1.3)
* M. szulgai*	39 (0.9)	7 (0.7)	10 (1.3)	12 (1.2)	10 (0.7)
* M. interjectum*	22 (0.5)	4 (0.4)	5 (0.6)	8 (0.8)	5 (0.4)
*M. terrae* group	18 (0.4)	6 (0.6)	3 (0.4)	5 (0.5)	4 (0.3)
* M. phocaicum/mucogenicum*	17 (0.4)	0	7 (0.9)	3 (0.3)	7 (0.5)
Other§	195 (4.7)	48 (5.1)	78 (9.8)	38 (3.7)	31 (2.3)
Clinical significance					
Isolation	905 (22)	216 (23)	212 (27)	202 (20)	275 (20)
Possible disease	1,060 (26)	111 (12)	132 (17)	258 (25)	559 (41)
Definite disease	2,158 (52)	621 (66)	449 (57)	567 (55)	521 (38)

*All values are no. (%). †Patients with samples from both pulmonary and extrapulmonary locations were categorized as having disseminated disease. ‡Species were grouped by using phylogenetic classifications described by Tortoli et al. (*7*). §Defined as *Mycobacteria* spp. and species with <15 cases reported throughout the study period.

**Figure 1:**
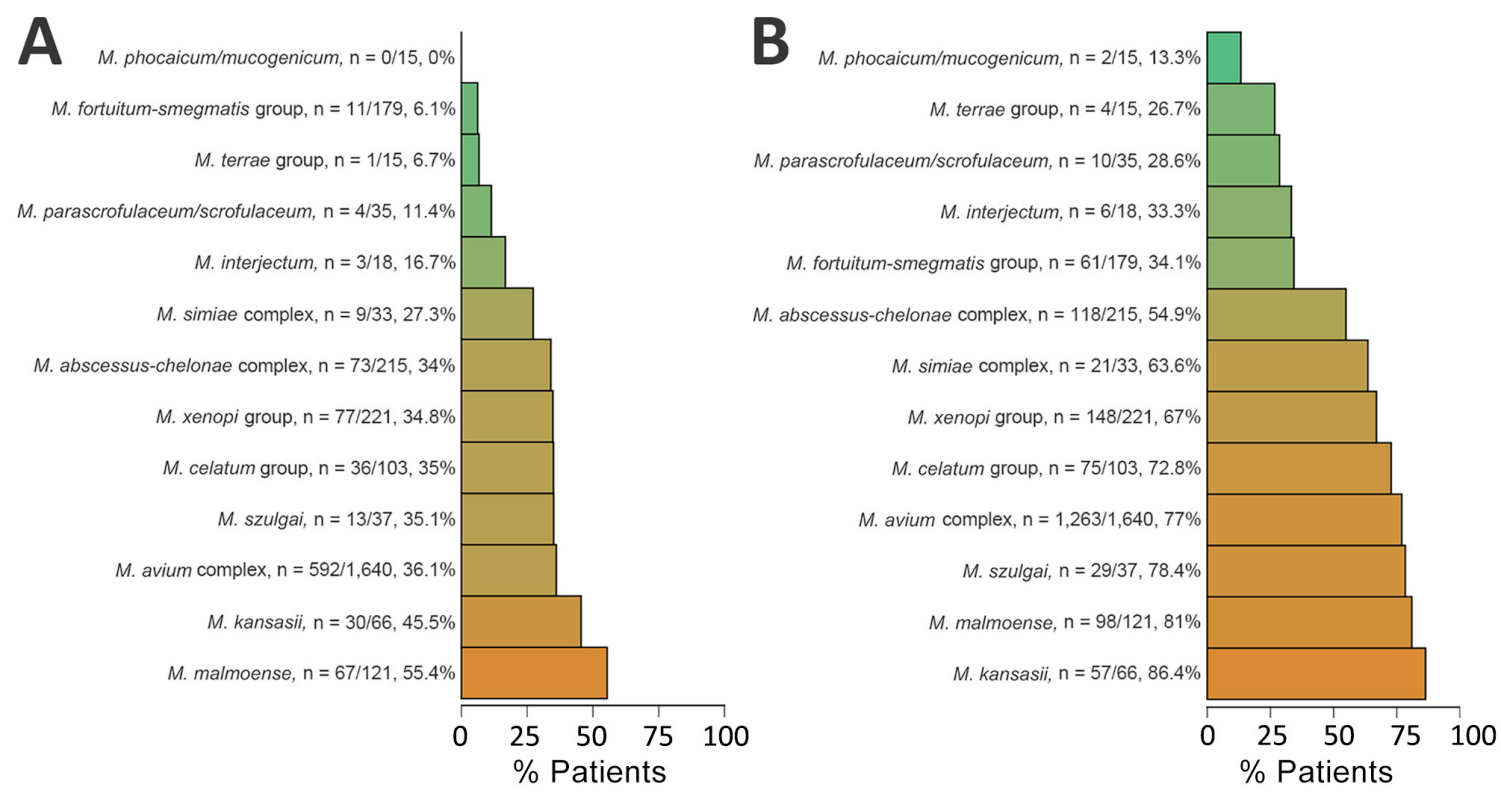
Absolute numbers and corresponding percentages of patients fulfilling modified criteria for nontuberculous mycobacteria pulmonary disease, based on microbiologic data only, Denmark, 1991–2022. A) Definite disease; B) definite and possible disease combined. Species were grouped by using phylogenetic classifications described by Tortoli et al. (*7*).

**Figure 2:**
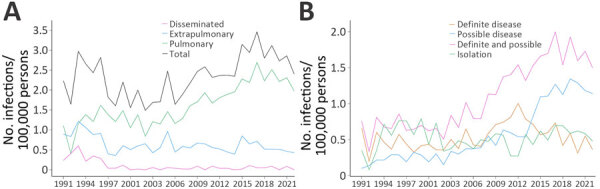
Annual incidence rates (infections/100,000 persons) of unique patients with a first culture positive for nontuberculous mycobacteria, by disease localization (A) and disease category for patients with pulmonary isolates only (B), Denmark, 1991–2022. Patients with samples from both pulmonary and extrapulmonary locations were categorized as having disseminated disease.

Approximately half of patients had definite disease (2,158 [52%]), whereas 26% (1,060) had possible disease and 22% (905) had isolation. One third of patients with pulmonary samples had definite disease (932 [33%]) and one third had possible disease (1,014 [36%]). We assessed clinical significance by species for patients with pulmonary samples (Figure 1). *M. malmoense* (55%), *M. kansasii* (46%), and MAC (36%) were most often associated with definite pulmonary NTM disease, whereas *M. kansasii* (86%), *M. malmoense* (81%), and *M. szulgai* (78%) were the most significant species when combing definite and possible disease. We also assessed the clinical significance of NTM isolation by species for any sample type and by age group and sex (Appendix Table 1, Figures 1, 2).

In all disease categories, MAC isolates were highly predominant, accounting for 68% (1,478/2,158) of definite disease cases and 65% (692/1,060) of possible disease cases but was slightly less common for isolation cases (42% [377/905]) (Appendix Table 1). *M. phocaicum*/*mucogenicum*, *M. parascrofulaceum*/*scrofulaceum*, and *M. terrae* group more often represented isolation cases. Disseminated NTM infections were mainly caused by MAC (87% [144/166]) (Appendix Table 2). All NTM species and groups were mostly isolated from pulmonary samples except for *M. marinum*, which was only found in extrapulmonary samples. *M. marinum* was the second-most common NTM isolated from extrapulmonary samples after MAC.

**Figure 3:**
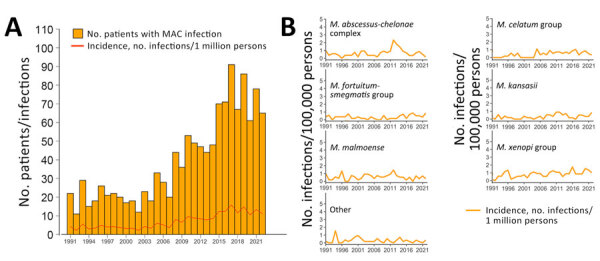
Annual absolute number of patients with a first culture positive for MAC, corresponding incidence rates, and annual incidence rates for the most frequent nontuberculous mycobacteria species for patients with definite and possible pulmonary disease combined, Denmark, 1991–2022. A) Numbers of first MAC cases by year and annual incidence (infections/1 million persons). B) Annual incidence rates (infections/1 million persons) of the most frequent nontuberculous mycobacteria species. Species were grouped by using phylogenetic classifications described by Tortoli et al. (*7*). Other was defined as *Mycobacteria* spp. and species with <15 cases reported throughout the study period. MAC, *Mycobacterium* *avium* complex.

**Figure 4:**
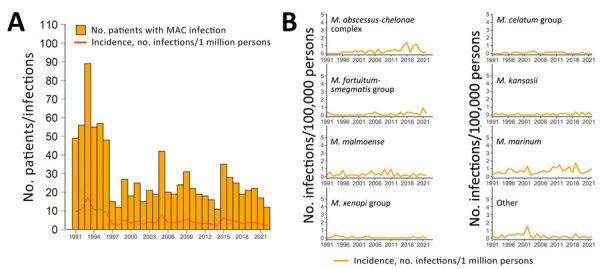
Annual absolute number of patients with a first culture positive for MAC, corresponding incidence rates, and annual incidence rates for the most frequent nontuberculous mycobacteria species for extrapulmonary and disseminated nontuberculous mycobacteria, Denmark, 1991–2022. Patients with samples from both pulmonary and extrapulmonary locations were categorized as having disseminated disease. A) Numbers of first MAC cases by year and annual incidence (infections/1 million persons). B) Annual incidence rates (infections/1 million persons) of the most frequent nontuberculous mycobacteria species. Species were grouped by using phylogenetic classifications described by Tortoli et al. (*7*). Other was defined as *Mycobacteria* spp. and species with <15 cases reported throughout the study period. MAC, *Mycobacterium* *avium* complex.

After adjustment for age and sex group differences, annual IRs of a positive NTM culture per 100,000 persons increased throughout the period (0.8%/year; p<0.001). This increase was driven mainly by an increase in patients with pulmonary NTM (2.3%/year; p<0.001), whereas the rate of extrapulmonary (−1.7%/year; p<0.001) and disseminated NTM (−9.5%/year; p<0.001) decreased (Figure 2, panel A). For pulmonary NTM, we also calculated annual IRs by disease categories (Figure 2, panel B). Annual IRs of patients with definite and possible pulmonary MAC increased over time (4.6%/year; p<0.001) (Figure 3), whereas the incidence of extrapulmonary and disseminated MAC decreased (Figure 4). These changes were mainly caused by subspecies *M. avium* (Appendix Figure 3). Infections attributable to *M. celatum* group (4.9%/year; p<0.001), *M. kansasii* (3.8%/year; p = 0.012), and *M. xenopi* (2.2%/year; p = 0.0152) also increased over time for definite and possible pulmonary NTM combined. The annual species distribution was comparable throughout the study period with discrete variations (Table).

## Discussion

Using nationwide mycobacteriologic data from Denmark during a 32-year period, we found that 52% of patients in whom NTM were isolated had clinically significant disease (i.e., definite); that percentage increased to 78% when possible disease was included. Species most often associated with definite NTM pulmonary disease, on the basis of microbiologic findings, were *M. malmoense*, *M. kansasii*, and MAC, whereas *M. kansasii*, *M. malmoense*, and *M. szulgai* were the most clinically significant species when possible disease was included as a sensitivity analysis. MAC infections, mainly attributable to *M. avium*, were predominant across disease categories and localizations, underscoring its status as the clinically most important species. However, the considerable differences in clinical significance and epidemiology identified in our study highlight that NTM should not be considered a single entity. We observed that pulmonary NTM incidence has been increasing in Denmark during the study period, in contrast to earlier reports (*4*,*5*).

Comparable to our findings, a previous study from Denmark that included data from a clinical survey found that approximately half of pulmonary NTM patients (31/58 [53.4%]) received treatment with >2 antimycobacterial drugs or were considered to have clinically significant NTM infection on the basis of signs and symptoms (*8*). Slightly less than half of those patients (39/85 [45.9%]) fulfilled clinical, radiologic, and microbiologic criteria for disease for those that had information available on all criteria.

Identifying NTM species, including to subspecies level, is crucial for selection of a treatment regimen and because different species are associated with varying clinical significance and outcomes (i.e., severity) (*9*–*11*). Several studies have investigated the clinical significance of NTM species (*12*–*20*), predominantly smaller studies with clinical data, whereas the larger studies have been based on laboratory data without clinical information. Many of those laboratory studies use the microbiologic component of the international guide criteria by the American Thoracic Society, European Respiratory Society, European Society of Clinical Microbiology and Infectious Diseases, and Infectious Diseases Society of America (*3*,*17*,*21*–*24*). Jankovic et al. found the positive predictive value of the microbiologic component compared with the full criteria was 59.8% (*17*); however, that value increased to 93.3% when a stricter definition was applied (*25*). Because many epidemiologic NTM studies use different approaches for defining NTM infection and disease (*26*), a consensus for reporting surveillance data is warranted to enable fair comparisons across countries and regions.

In our study, we applied stricter criteria than those used in many other studies, enabling comparisons with previous studies (*4*,*5*). Those criteria have been shown to have high positive predictive values for clinically significant disease (*4*). We found that 33% of patients with pulmonary NTM had definite disease, comparable to the number of patients fulfilling American Thoracic Society and Infectious Diseases Society of America criteria in other studies, albeit slightly higher (*12*,*15*,*20*). A previous study of pulmonary NTM in eastern Asia found that 31% (582/1,744) of patients fulfilled those criteria (*16*). In studies from northwestern Europe, *M. kansasii*, *M. szulgai*, and *M. malmoense* have been associated with a high degree of clinical significance (>70%) (*27*,*28*). In other regions of the world, those species are considered less clinically significant, demonstrating that clinical significance varies with geography.

MAC was the most predominant group of pulmonary NTM (58%) in Denmark (*5*). In contrast, *M. xenopi* (7.8%) and *M. kansasii* were rare (2.3%) compared with findings in eastern and southern Europe, whereas *M. malmoense* was much more incident (4.2%) than in countries outside Scandinavia (*29*,*30*). *M. abscessus-chelonae* complex accounted for the second-most frequently observed NTM group (7.5%). We observed clear differences in trends over time for the different species. For instance, the incidence of *M. malmoense* has previously been shown to rise in northern Europe (*31*), and although we did not observe an increase (–1.3%/year; p = 0.113), we could confirm the relative significance in our region, finding similar proportions of clinical significance (up to 81%) as reported in a systematic review of clinical data (70%–80%) (*31*). The abundance of *M. malmoense* in northern Europe, compared with southern Europe (0.6% of 3,696 isolates) and rest of the world (1% of ≈20,000 isolates), remains unexplained (*30*). The most extensive evaluations of geographic differences in the distribution of NTM species are older (*30*,*32*), and because NTM epidemiology is clearly changing over time, reevaluations of geographic differences are necessary.

Until recently, no increasing trends in NTM incidence had been reported in Denmark (*4*,*5*), in contrast to most other parts of the world (*33*). However, recent studies based on diagnostic codes from the International Classification of Diseases, 10th Revision, a potential proxy for NTM-treated patients, also found that NTM incidence and prevalence have been increasing in Denmark (*34*,*35*). Again, those findings show that temporal and geographic differences are essential when evaluating NTM epidemiology, underlining the need for surveillance. Diagnostic codes may have a lower sensitivity but a good positive predictive value (*36*), suggesting that combining the different approaches could be useful. Our study, showing an increase of pulmonary NTM incidence over time but not of extrapulmonary and disseminated NTM, suggests that structural lung disease and advancing age could be among the main determinants for those changes. This hypothesis is supported by the fact that structural lung disease is the strongest risk factor for NTM pulmonary disease (*37*). Still, sampling for NTM from pulmonary sites probably evolved over time, especially with increasing awareness of chronic lung conditions predisposing to NTM, whereas sampling from extrapulmonary sites is mainly based on disease manifestations (*37*). Hence, in the past decade, or even longer, patients living with chronic lung disease might have had cultures examined regularly as part of standard of care, leading to a higher number of positive NTM cultures. Further discussion of potential explanations for the increasing trends is available elsewhere (*1*,*33*).

One limitation of our study is the lack of clinical information, including data on symptoms, underlying conditions, risk factors for NTM, radiologic findings, and treatment. Although the use of microbiologic data as a measure of clinical significance may be flawed, lacking clinical information and a description of sampling strategy, we believe microbiologic data are a useful proxy for NTM disease and a valuable tool for NTM surveillance (*33*).

In conclusion, *M. malmoense*, *M. kansasii*, *M. szulgai*, and MAC are the most clinically significant NTM in Denmark based on microbiologic findings, with *M. avium* being of greatest importance because of its dominance in incidence. In contrast to earlier findings, the incidence of pulmonary NTM has been increasing in Denmark. This study shows the critical need for surveillance for an emerging infection that is not notifiable in most countries, provides evidence to support clinical decision-making, and highlights the importance of not considering NTM as a single entity.

Appendix Additional information about clinical significance, species distribution, and temporal trends of nontuberculous mycobacteria, Denmark, 1991–2022.
