# Supplementary material for: Clinical Significance, Species Distribution, and Temporal Trends of Nontuberculous Mycobacteria, Denmark, 1991–2022
Source: Emerg Infect Dis. 2024 Sep;30(9):1755–62. doi: 10.3201/eid3009.240095 (PMC11346989; doi:10.3201/eid3009.240095)
Supplement: Appendix — Additional information about clinical significance, species distribution, and temporal trends of nontuberculous mycobacteria, Denmark, 1991–2022. [file 24-0095-Techapp-s1.pdf]

Article DOI: https://doi.org/10.3201/eid3009.240095

EID cannot ensure accessibility for supplementary materials supplied by authors. Readers who have difficulty accessing supplementary content should contact the authors for assistance.

Clinical Significance, Species Distribution, and Temporal Trends of Nontuberculous Mycobacteria, Denmark, 1991–2022

Appendix

Appendix Table 1. Species distribution of nontuberculous mycobacteria by disease categories* in Denmark from 1991–2022.

Species**	Definite disease, n (%)	Possible disease, n (%)	Isolation, n (%)
M. avium complex	1,478 (58)	692 (27)	377 (15)
M. avium	1,296 (63)	482 (24)	268 (13)
M. intracellulare	130 (39)	137 (41)	67 (20)
M. chimaera	38 (32)	52 (44)	29 (24)
Other/unspecified	14 (29)	21 (44)	13 (27)
M. abscessus-chelonae complex	132 (48)	46 (17)	97 (35)
M. xenopi group	86 (37)	72 (31)	73 (32)
M. fortuitum-smegmatis group	31 (15)	54 (27)	117 (58)
M. malmoense	114 (68)	31 (18)	23 (14)
M. marinum	128 (100)	0	0
M. celatum group	47 (40)	42 (36)	28 (24)
M. kansasii	45 (56)	27 (33)	9 (11)
M. simiae complex	19 (44)	12 (28)	12 (28)
M. parascrofulaceum/scrofulaceum	8 (20)	7 (18)	25 (62)
M. szulgai	15 (38)	16 (41)	8 (21)
M. interjectum	6 (27)	4 (18)	12 (55)
M. terrae group	3 (17)	4 (22)	11 (61)
M. phocaicum/mucogenicum	2 (12)	2 (12)	13 (76)
Other***	44 (23)	51 (26)	100 (51)
Total, n = 4123 (%)	2158 (52)	1060 (26)	905 (22)

*See methods for criteria.
**Species were grouped using phylogenetic classifications described by Tortoli et al. (*1*).
***Defined as *Mycobacteria* spp. and species with n < 15 throughout the study period.

Appendix Table 2. Species distribution of nontuberculous mycobacteria by disease localization in Denmark from 1991–2022.

Species*	Pulmonary, n (%)	Extra-pulmonary, n (%)	Disseminated, n (%)**
M. avium complex	1,640 (58)	763 (69)	144 (87)
M. avium	1,172 (57)	735 (36)	139 (6.8)
M. intracellulare	315 (94)	17 (5.1)	2 (0.6)
M. chimaera	115 (97)	2 (1.7)	2 (1.7)
Other/unspecified	38 (79)	9 (19)	1 (2.1)
M. abscessus-chelonae complex	215 (78)	54 (20)	6 (2.2)
M. xenopi group	221 (96)	6 (2.6)	4 (1.7)
M. fortuitum-smegmatis group	179 (89)	22 (11)	1 (0.5)
M. malmoense	121 (72)	46 (27)	1 (0.6)
M. marinum	0	128 (100)	0
M. celatum group	103 (88)	13 (11)	1 (0.9)
M. kansasii	66 (81)	10 (12)	5 (6.2)
M. simiae complex	33 (77)	8 (19)	2 (4.7)
M. parascrofulaceum/scrofulaceum	35 (88)	4 (10)	1 (2.5)
M. szulgai	37 (95)	2 (5.1)	0
M. interjectum	18 (82)	4 (18)	0
M. terrae group	15 (83)	3 (17)	0
M. phocaicum/mucogenicum	15 (88)	2 (12)	0
Other***	153 (78)	41 (21)	1 (0.5)
Total, n = 4123 (%)	2851 (69)	1106 (27)	166 (4)

*Species were grouped using phylogenetic classifications described by Tortoli et al. (*1*).

**Patients with samples from both pulmonary and extra-pulmonary locations were categorized as disseminated disease.

***Defined as *Mycobacteria* spp. and species with n < 15 throughout the study period.

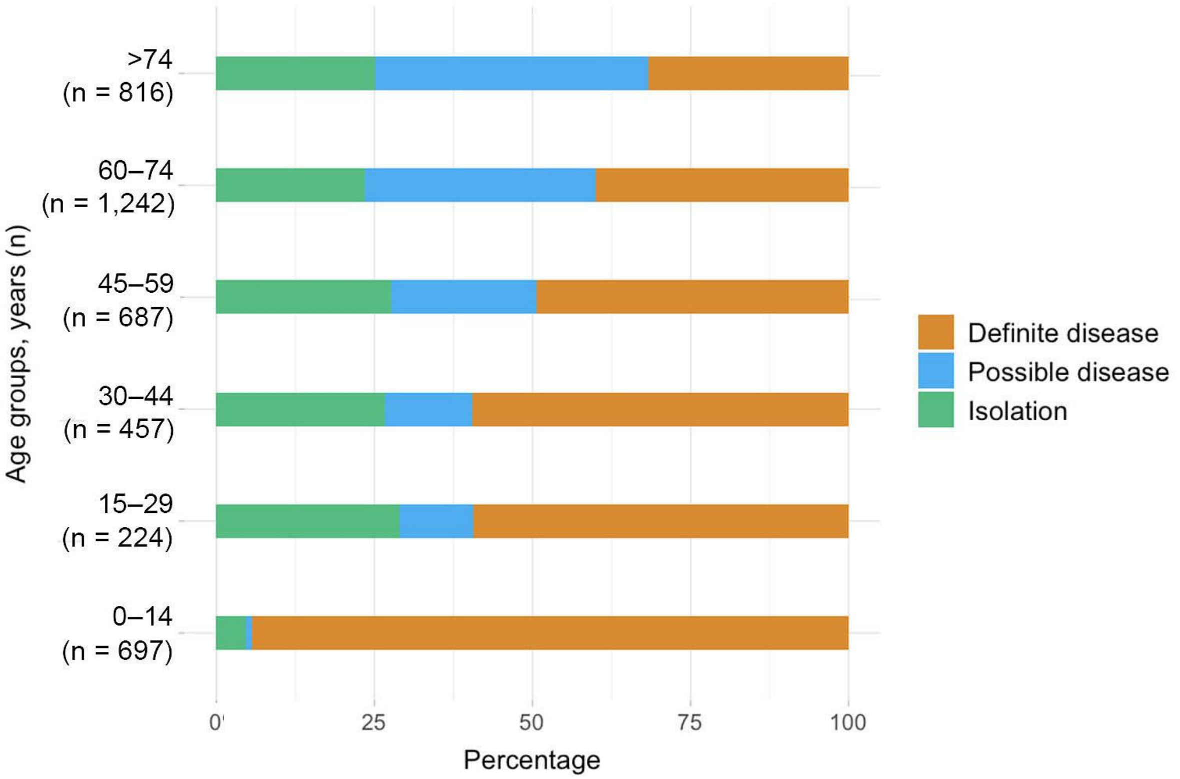

Appendix Figure 1. Clinical significance of nontuberculous mycobacteria by age groups in Denmark from 1991–2022. See methods for criteria.

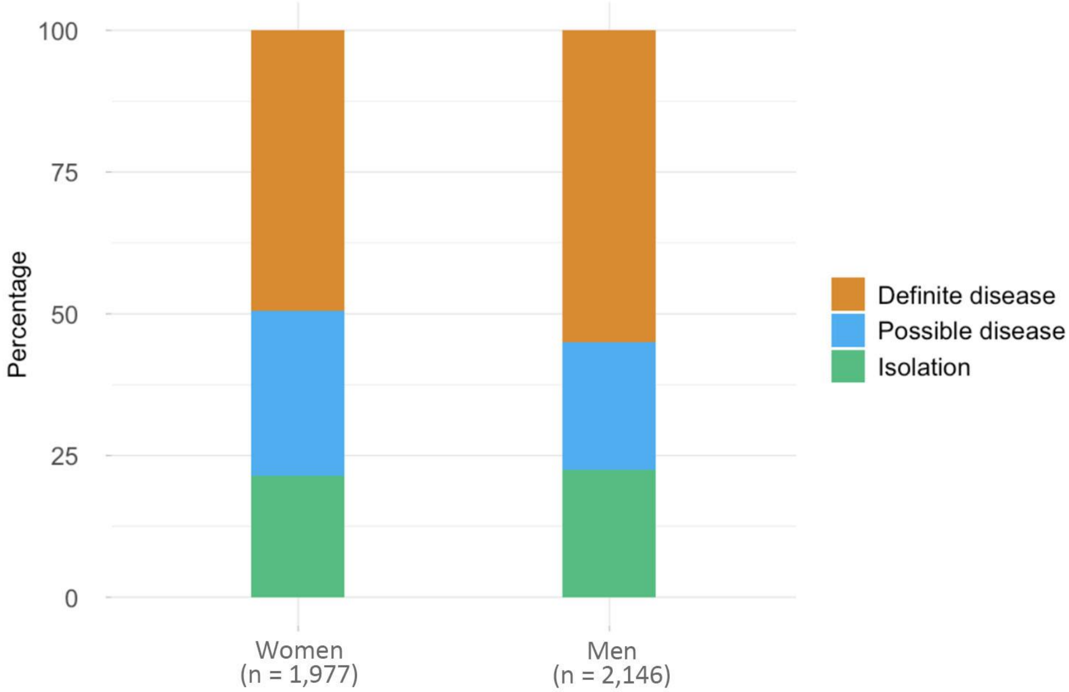

Appendix Figure 2. Clinical significance of nontuberculous mycobacteria by sex in Denmark from 1991–2022. See methods for criteria.

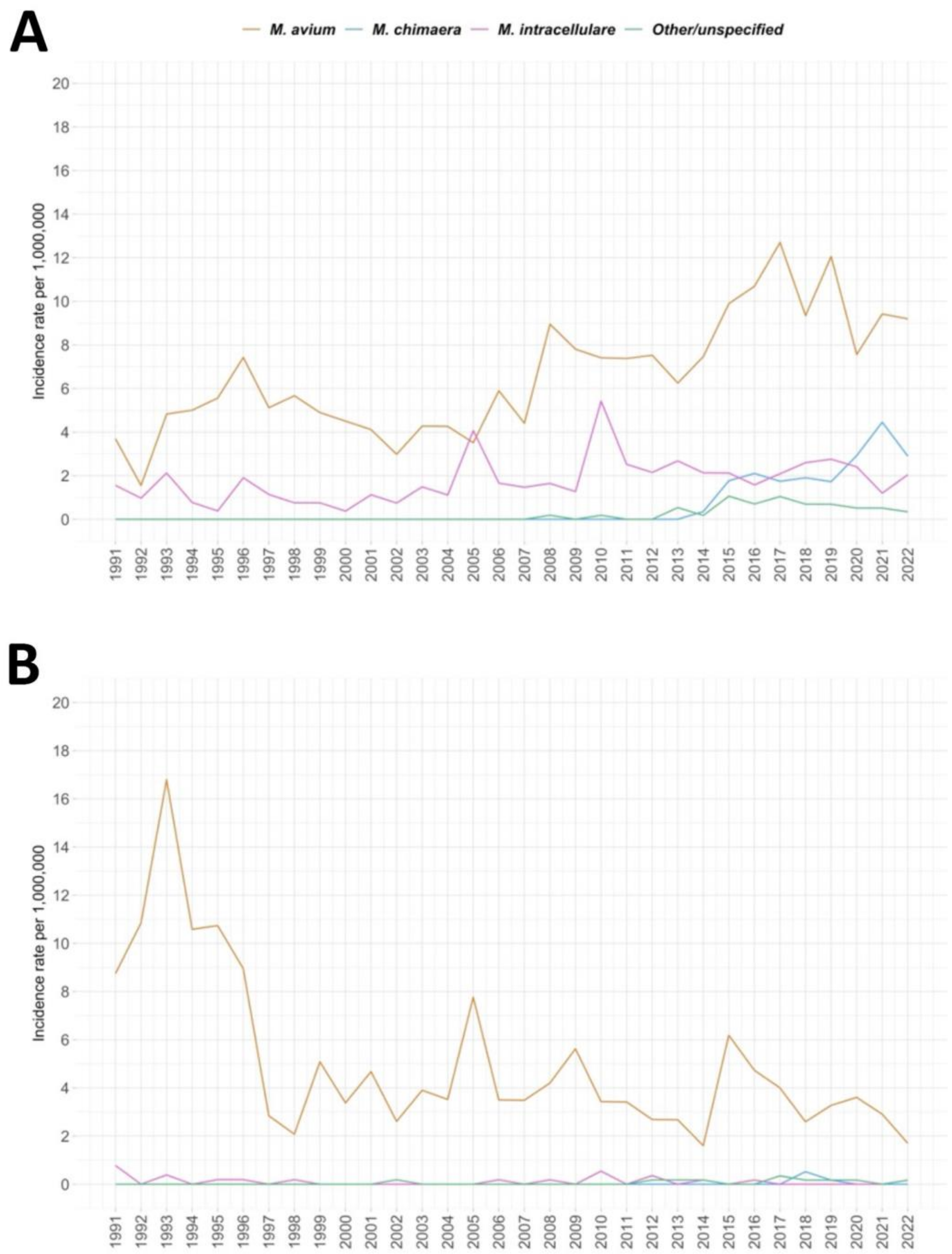

Appendix Figure 3. Annual incidence rates (infections/1,000,000 persons) of unique patients with a first culture positive for *M. avium* complex Denmark from 1991–2022 by subspecies for patients with pulmonary (Panel A) and extrapulmonary and disseminated nontuberculous mycobacteria (Panel B). Patients with samples from both pulmonary and extrapulmonary locations were categorized as disseminated disease. Species were grouped using phylogenetic classifications described by Tortoli et al. (*1*).

Reference

1. Tortoli E, Fedrizzi T, Meehan CJ, Trovato A, Grottola A, Giacobazzi E, et al. The new phylogeny of the genus *Mycobacterium*: the old and the news. Infect Genet Evol. 2017;56:19–25. PubMed https://doi.org/10.1016/j.meegid.2017.10.013
